# *Molecules* Best Paper Award 2012

**DOI:** 10.3390/molecules17021795

**Published:** 2012-02-10

**Authors:** Derek J. McPhee

**Affiliations:** Editor-in-Chief, MDPI AG Postfach, CH-4005 Basel, Switzerland; Email: mcphee@mdpi.com

*Molecules *has started to institute a “Best Paper” award to recognize the most outstanding papers in the area of natural products, medicinal chemistry and molecular diversity published in Molecules. We are pleased to announce the first “*Molecules* Best Paper Award” for 2012. Nominations were selected by the Editor-in-Chief and selected editorial board members from among all the papers published in 2008. Reviews and research papers were evaluated separately. We are pleased to announce that the following three papers have won the Molecules Best Paper Award in 2012: 

## Article Award:

### 1^st^ Prize

**Sangmin Kim, Jae Hyuck Choi, Jong Bin Kim, Seok Jin Nam, Jung-Hyun Yang, Jung-Han Kim and Jeong Eon Lee**

Berberine Suppresses TNF-α-induced MMP-9 and Cell Invasion through Inhibition of AP-1 Activity in MDA-MB-231 Human Breast Cancer Cells 

*Molecules ***2008**, *13*(12), 2975-2985; doi:10.3390/molecules13122975

Available online: http://www.mdpi.com/1420-3049/13/12/2975/

### 2^nd^ Prize

**Faustine Dubar, Jamal Khalife, Jacques Brocard, Daniel Dive and Christophe Biot**

Ferroquine, an Ingenious Antimalarial Drug –Thoughts on the Mechanism of Action 

*Molecules ***2008**, *13*(11), 2900-2907; doi:10.3390/molecules13112900 

Available online: http://www.mdpi.com/1420-3049/13/11/2900/

## Review Award:

### 1^st^ Prize

**Masakiyo Hosokawa**

Structure and Catalytic Properties of Carboxylesterase Isozymes Involved in Metabolic Activation of Prodrugs

*Molecules ***2008**, *13*(2), 412-431; doi:10.3390/molecules13020412

Available online: http://www.mdpi.com/1420-3049/13/2/412/

We believe these three exceptional papers represent valuable contributions to *Molecules* and the scientific literature. On behalf of the Prize Awarding Committee and the Editorial Board of *Molecules*, we would like to congratulate these three teams for their excellent work. In recognition for their accomplishment, Drs. Jung-Han Kim and Jeong Eon Lee, Dr. Christophe Biot and Dr. Masakiyo Hosokawa will receive prizes of 1000 CHF, 500 CHF, and 500 CHF, respectively, and the privilege of publishing an additional of their choice paper free of charge in Open Access format in *Molecules, *after the usual peer-review procedure. 

### Prize Awarding Committee

*Editor-in-Chief*

**Dr. Derek J. McPhee**

MDPI AG Postfach, CH-4005 Basel, Switzerland

Email: mcphee@mdpi.com

*Editorial Board Member*, Section ‘Natural Products”

**Dr. John A. Beutler**

Molecular Targets Laboratory, Bldg 1052 Rm 110 NCI at Frederick, Frederick, MD 21702-1201, USA

Email: beutlerj@mail.nih.gov

*Editorial Board Member*, Section ‘Medicinal Chemistry’

**Prof. Dr. Jarkko Rautio**

School of Pharmacy, University of Eastern Finland, P.O.Box 1627, 70211 Kuopio, Finland

Email: jarkko.t.rautio@uef.fi

*Editorial Board Member*, Section ‘Organic Synthesis’

**Prof. Dr. Osmo E. O. Hormi**

Department of Chemistry, University of Oulu, Linnanmaa, P.O.Box 333, FIN-90570 Oulu, Finland

Email: osmo.hormi@oulu.fi

*Editorial Board Member*, Section ‘Molecular Diversity’

Dr. Shu-Kun Lin 

MDPI AG, Postfach, CH-4005 Basel, Switzerland. Office: Kandererstrasse 25, 4057 Basel, Swizerland

Email: lin@mdpi.com

